# Modeling of the Small-Scale Outbreak of COVID-19

**DOI:** 10.3389/fpubh.2022.907814

**Published:** 2022-07-01

**Authors:** Ze-Yang Wu, Hong-Bo Zhang, Hong-Fei Zhao

**Affiliations:** ^1^Department of Digital Media Technology, College of Computer Science and Technology, Huaqiao University, Xiamen, China; ^2^Fujian Key Laboratory of Big Data Intelligence and Security, Huaqiao University, Xiamen, China; ^3^Faculty of Science, National University of Singapore, Singapore, Singapore

**Keywords:** COVID-19, small-scale outbreak, cellular automata, time matrix, simulation

## Abstract

With the improvement of treatment and prevention methods, many countries have the pandemic under control. Different from the globally large-scale outbreak of COVID-19 in 2020, now the outbreak in these countries shows new characteristics, which calls for an effective epidemic model to describe the transmission dynamics. Meeting this need, first, we extensively investigate the small-scale outbreaks in different provinces of China and use classic compartmental models, which have been widely used in predictions, to forecast the outbreaks. Additionally, we further propose a new version of cellular automata with a time matrix, to simulate outbreaks. Finally, the experimental results show that the proposed cellular automata could effectively simulate the small-scale outbreak of COVID-19, which provides insights into the transmission dynamics of COVID-19 in China and help countries with small-scale outbreaks to determine and implement effective intervention measures. The countries with relatively small populations will also get useful information about the epidemic from our research.

## 1. Introduction

In December 2019, the high-speed expansion of COVID-19 managed itself into a global pandemic in a minute, which ended up as a global crisis. China launches a resolute battle to prevent and control the spread of COVID-19, within 4 months the transmission of the virus has been successfully cut off. The daily confirmed cases in China mainland dropped below 100 and further declined to a single digit. Hard work of China had gained remarkable achievement. Unfortunately, the virus has mutated in a way that might spread easier, which poses a great challenge to epidemic prevention.

**Figure 1:**
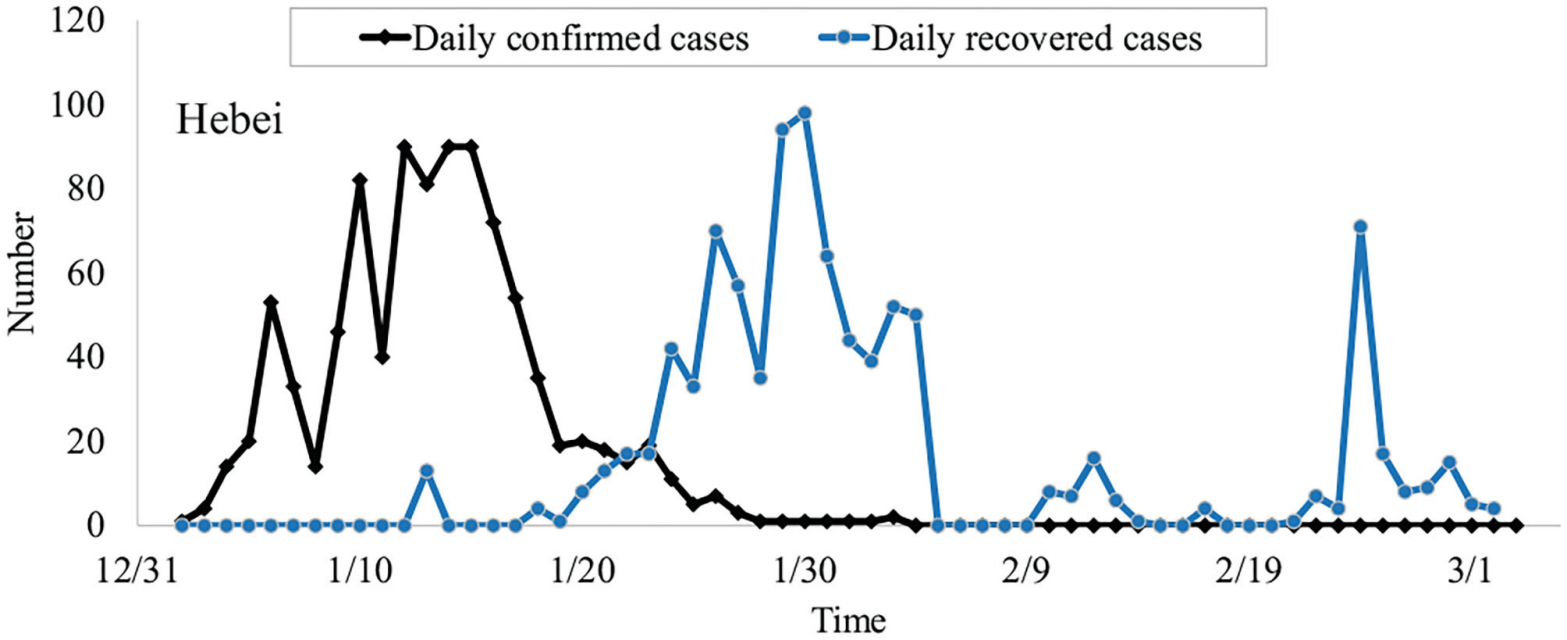
The daily confirmed cases and recovered cases of COVID-19 in Hebei province on January 2021.

According to the data from the National Health Commission of China, we can obtain some general principles underlying the spread of the virus, as shown in Figure 1. During outbreaks, the daily confirmed cases are less than one hundred. The outbreaks will last for around 30 days. It is noticeable that the trend of daily confirmed cases reached its peak around 15 days after the outbreak and the daily new recovered cases peak at 20 days later.

With the experience of fighting against COVID-19 in Wuhan, the Chinese government has had science-based measures for COVID-19 prevention and control. In this article, we set our attention on the small-scale outbreak and transmission of COVID-19 in various provinces of China, and try to reveal the general principles underlying the spread of the virus to provide theoretical support for epidemic prevention. With appropriate parameter setting and transmission rules, this study can also be used for epidemic analysis in many other countries. The stability of the proposed model is tested with COVID-19 data in Potter County, Texas US. This study can provide important information for making appropriate decisions in countries that lack medical resources.

Many models can be applied to simulate the spread of the epidemic. The traditional SIR model has already been widely used to simulate the epidemic at a country level, generally with numerous infected cases a huge population (1–4). Nowadays, many countries already have the COVID-19 under control, and the infected cases in a city or county with a relatively small population, are marginal compared with the total infected cases at the country level. Most of the previous studies focused their attention on outbreaks with sufficient infected cases, like the outbreak in Wuhan (4, 5). We believe that COVID-19 will show a trend of small outbreaks within a certain range. It is significant to explore the regularity of small-scale outbreaks of COVID-19. In this study, we focus our attention on small-scale outbreaks with a limited number of cases. First, we applied traditional SIR model and *SEI*_*U*_*I*_*D*_*R*_*U*_*R*_*D*_ model simplified from *SEI*_*D*_*I*_*U*_*QHRD* (6). Then a new version of CA is proposed to carry out the experiment. In the improved CA model, the parameters and transmission rules are set according to the data from the local health department. During the experiment, we simulate the outbreaks in two provinces of China, Heilongjiang and Hebei, and Potter County, Texas US. The results show that our improved CA has a better performance compared with the two compartments models mentioned above.

The contribution of this study can be summarized as follows. To the best of our knowledge, this is the first study that tries to simulate the small-scale outbreak of COVID-19. Additionally, a new version of CA has been proposed with a time matrix that can simulate the outbreak well by setting transmission rules. Utilizing this model, epidemic trends in small-scale outbreaks can be used to help health officials make decisions on public health policies.

## 2. Related Study

In 2002, the SARS (Severe Acute Respiratory Syndrome) virus was first found in Guangdong China. Classical compartmental models SIR have been used in simulation and prediction (7–9). In their studies, the number of susceptible, infected, and recovered from Beijing have been calculated using the SIR model, and all parameters with epidemiological meaning including transmission rate, removal rate, and basic reproduction number have been estimated. The same methods have been used in investigating the transmission rules of SARS in Guangdong province (10). The studies showed that transmission dynamic models, in the form of differential equations, could simulate the process of SARS transmission with reasonable parameters and reflect the dynamic of SARS transmission.

Since December 2019, the COVID-19 started its transmission, and classical compartmental models have been widely used in predictions. But the rate of transmission and many other parameters in classical models are constants. For better simulation, numerous researchers have proposed many methods of predicting the parameters dynamically (7, 11, 12). The improved SEIR model has been used in forecasting the outbreak and combined with a series of interventions formulated by the government. With the development of machine learning, a dynamic prediction method of the infection rate was derived based on long short-term memory (LSTM) and has a better performance compared with that of the traditional SEIR model (13, 14). These models assumed that populations are completely mixed and ignore spatial effects of spread epidemics; also interaction between individuals is neglected since they model populations as continuous entities (15).

Cellular automata are dynamic systems with discrete time, space, and state. It discusses the overall properties on the premise of synchronous updating based on local principles, which is expected to simulate the real epidemic situation through the set of local principles (16). It has been applied in the field of infectious disease control (17–19). Classical epidemic models based on differential equations may be unsuitable for simulating small-scale outbreaks of COVID-19, given the lack of flexibility when simulating local characteristics of infectious diseases. Cellular automata may have better performance in the simulation of small-scale virus outbreaks.

In previous studies (13, 20), most of the methods have been directed at the large-scale outbreak of SARS or COVID-19. Most predictive studies based on cellular automata focused on H1N1 and the small spread of chickenpox (19, 21). At present, the epidemic situation in China is generally stable, with rebounds in some provinces. In this contribution, we used classic compartmental models (SIR and *SEI*_*U*_*I*_*D*_*R*_*U*_*R*_*D*_) and CA to simulate the small-scale outbreaks of COVID-19 in different provinces of China, a time matrix is set to optimize the cellular automata.

Alongside CA, many researchers have implemented an agent-based model (ABM) in simulating the pandemic (22, 23). ABM to some extent evolved from CA, they are a class of agents, and each of them contains variable information. Each agent can interact with their neighbors and transform their state. The major difference between ABM and CA is that each agent within ABM can move their position as well as change state and interact with neighbors. However, the cell in the class of CA will not be able to transform their physic position. ABM is more intuitive than mathematical or statistical models because it represents objects as individual things in the world. In previous studies, ABM models have been used in searching for cost-effective proactive testing strategies and simulating the effects of health policy (24, 25).

## 3. Methods

### 3.1. SIR Model

In the SIR model, individuals are assigned to three compartments or categories: susceptible(S), infectious(I), and recovered(R). S compartment represents the susceptible individuals that are not immune to the virus and might get infected when exposed to it. I compartment stands for those individuals who are carrying the virus and can spread it. R compartment indicates those infected with the virus and have successfully recovered after treatment or died. Suppose that the recovered individuals will not be re-infected or spread the virus.

**Figure 2:**
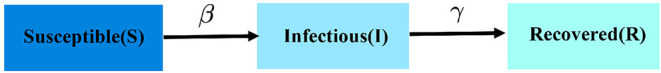
The framework of SIR model.

As a result of the China's public health emergency system and strict traffic controls, the number of deaths is close to zero and population migration with neighboring provinces is negligible. It is reasonable to suppose that the population remains constant during the outbreak, and the birth, death, and migration rates are zero. SIR model can be described by the following set of differential equations.


(1) $$\begin{array}{r} \begin{array}{cc} \frac{dS}{dt} & =-\beta \frac{S}{N}I\\ \frac{dI}{dt} & =\beta \frac{S}{N}I-\gamma I\\ \frac{dR}{dt} & =\gamma I\\ N & =S\left(t\right)+I\left(t\right)+R\left(t\right) \end{array} \end{array}$$

where *N* is the total population of an area and it remains a constant during the spread of the virus and *S*(*t*), *I*(*t*), *R*(*t*) represent the number of individuals in a different compartment at the time *t*. β is the infection rate, which means the transition probability from *S* to *I*. Similarly, γ is the removal rate, which represents the transition probability from *I* to *R*. They are often regarded as constants for simplicity of calculation. $\frac{dS}{dt}$ is the changing rate of susceptible individuals. The number of susceptible individuals decreases with the increment of infected individuals. $\frac{dI}{dt}$ is the changing rate of infected individuals. $\frac{dR}{dt}$ is the changing rate of recovered individuals. The framework of SIR is shown in Figure 2.

### 3.2. *SEI*_*U*_*I*_*D*_*R*_*U*_*R*_*D*_ Model

Based on the SIR model, we further analyzed China's epidemics prevention and set up the SEIR model. Due to the characteristic of COVID-19, there will be a latency when individuals are exposed to the virus (9). During the latency, exposed individuals are incapable of transmitting the virus and the illness did not deteriorate to the infected stage. Susceptible individuals may become exposed. Exposed individuals (E) will eventually evolve into the infected. Therefore, after being exposed to the virus, patients usually turn into I after latency. However, in SIR the exposed individual is not modeled. In addition, there are two types of infectious diseases: symptomatic infectious (*I*_*D*_) and asymptomatic infectious (*I*_*U*_) (6). Due to the strict prevention and control measures, a newly detected *I*_*D*_ will get a strict quarantine, and the transmission of the virus will be cut off. Consequently, the original SEIR model is extended to the *SEI*_*U*_*I*_*D*_*R*_*U*_*R*_*D*_ model. The *SEI*_*U*_*I*_*D*_*R*_*U*_*R*_*D*_ model can be described as follow.


(2) $$\begin{array}{r} \begin{array}{cc} \frac{dS}{dt} & =-\beta \frac{S\left(I_{U}+\sigma I_{D}\right)}{N}\\ \frac{dE}{dt} & =\beta \frac{S\left(I_{U}+\sigma I_{D}\right)}{N}-\eta E\\ \frac{dI_{U}}{dt} & =\phi \eta E-\gamma I_{U}\\ \frac{dI_{D}}{dt} & =\left(1-\phi \right)\eta E-\gamma I_{D}\\ \frac{dR_{U}}{dt} & =\gamma I_{U}\\ \frac{dR_{D}}{dt} & =\gamma {\text{I}}_{\text{D}} \end{array} \end{array}$$

**Figure 3:**
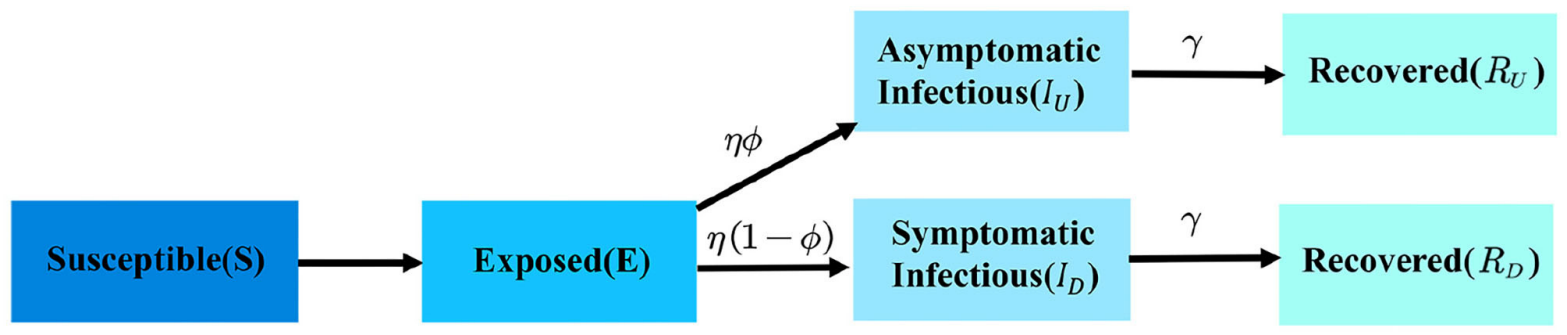
The framework of *SEI*_*U*_*I*_*D*_*R*_*U*_*R*_*D*_ model.

Compartments definition (Figure 3):

- Susceptible (S) is the part of the population that could be potentially subjected to the infection.
- Exposed (E) is the fraction of the population that has been infected but does not show symptoms yet: it can be called a latent phase. and at this stage, we define it to be not infectious.
- Asymptomatic Infectious (*I*_*U*_) is people infected with a novel coronavirus that does not exhibit symptoms at any time during the course of infection, and are capable of spreading the virus. They are a potential source of substantial spread within the community (6). Due to the undetectable character of *I*_*U*_, we assume all the infected cases collected by the health department are symptomatic infectious (*I*_*D*_).
- Symptomatic Infectious (*I*_*D*_) stands for population infected with the virus and exhibit a verity of symptoms: fever or chills, cough, shortness of breath, or difficulty breathing. Isolation is needed to cut off the spread of the virus according to the local health policies. In the later experiment, we assume that confirmed cases collected by the public health department represent *I*_*D*_ only.
- Undetected Recovered (*R*_*U*_) are the people healed from *I*_*U*_, they have become immune to the virus and will no be reintroduced into the susceptible category.
- Detected Recovered (*R*_*D*_) are the people healed from *I*_*D*_, similar to *R*_*U*_ they are immune to the virus, but stand for recovered cases that are in the health department record.

The framework of the *SEI*_*U*_*I*_*D*_*R*_*U*_*R*_*D*_ model is shown in Figure 3.

Parameters value definition:

(a) β infection rate. It is the number of people that a patient can infect each day, which transports people from the S category to the E category. We define it as a constant and the estimation is in the setting of a later parameter.
(b) η transform rate from E to *I*_*D*_ or *I*_*U*_, which represents the incubation and is defined as a constant.
(c) ϕ percentage of infections that are asymptomatic stands for the proportion of asymptomatic infectious individual (*I*_*U*_) in all infections. For example, a parameter value of 0.5 represents that half of the exposed population will transform into *I*_*U*_.
(d) σ the non-isolation rate of the symptomatic infectious individual (*I*_*D*_). In some countries, the σ is set to 0, which means that all symptomatic infectious are isolated and incapable of transmitting the virus.
(e) γ stands for recovery rate. It gives information about how fast people may recover from the disease or pass away during the treatment (1/γ is the average recovery time).

### 3.3. Cellular Automata

During the transmission, the relationships between data of infectious diseases are extremely complex. However, cellular automata can predict the epidemic through multi-step iteration and parallel evolution only by determining relatively simple evolution rules (18). In the case of traditional dynamics models that are unsuitable for modeling the spread of COVID-19 in China nowadays, we try to use cellular automata to carry out the experiment. Cellular automata are a dynamic system discrete in time, space, and state, different kinds of cells represent different groups of people: S-cell represents susceptible, E-cell represents exposed, *I*_*U*_-cell is asymptomatic infectious, *I*_*D*_-cell is symptomatic infectious, *R*_*U*_-cell is Undetected recovered, *R*_*D*_-cell the detected recovered. The transmission rules of COVID-19 in cellular automata are the same as *SEI*_*U*_*I*_*D*_*R*_*U*_*R*_*D*_.

When cellular automata are used to simulate the transformation from the Infected (*I*_*U*_*andI*_*D*_) to Recovered (*R*_*U*_*andR*_*D*_), previous studies often generate a random number and make a comparison with the removal rate, γ. If the random number is less than γ, the infected cell will turn into recovered (1). However, it is not satisfactory for the real scene. Therefore, we introduce a time matrix to record the time of virus infection of each cell as defined in Equation (3).


(3) $$T_{n\times n}=\left[\begin{array}{cccc} t_{11} & t_{12} & \ldots & t_{1n}\\ t_{21} & t_{22} & \ldots & t_{2n}\\ \vdots & \vdots & \ddots & \vdots \\ t_{n1} & t_{n2} & \ldots & t_{nn} \end{array}\right]$$

At first, all elements in the time matrix, *T*_*n* × *n*_, are set to 0. The state of the cell is 0 and there is a 0.5% probability of a cell turning into 1, which represents exposure at the beginning of the outbreak. The structure of CA will be defined as follows:

- The two dimensional lattice of square cells in an orthogonal grid. The size of the orthogonal grid is *n* × *n*, a vector (*i, j*) represents the position of the cell in the grid.
- The size of the grid is theoretical infinity, but in the experiment, we set it as *n*^2^ = 300^2^. Each cell's neighborhood is composed of all its eight neighboring cells (the Moore neighborhood).
- Each cell has six states, we can picture 0 as the state of being susceptible to a given cell, 1 as the state of being exposed, 2 as being asymptomatic infectious (*I*_*U*_), 3 as symptomatic infectious individuals (*I*_*D*_) that are **not** being isolated, 4 as symptomatic infectious individuals (*I*_*D*_) that are being isolated, last 5 and 6 as *R*_*U*_ and *R*_*D*_, respectively.
- The time matrix *T* as defined in the previous section will record the time when cells turn into the Exposed. COVID-19's transition rule goes as follows. At each time step *t* exactly one of five things can happen to a cell.

**Figure 4:**
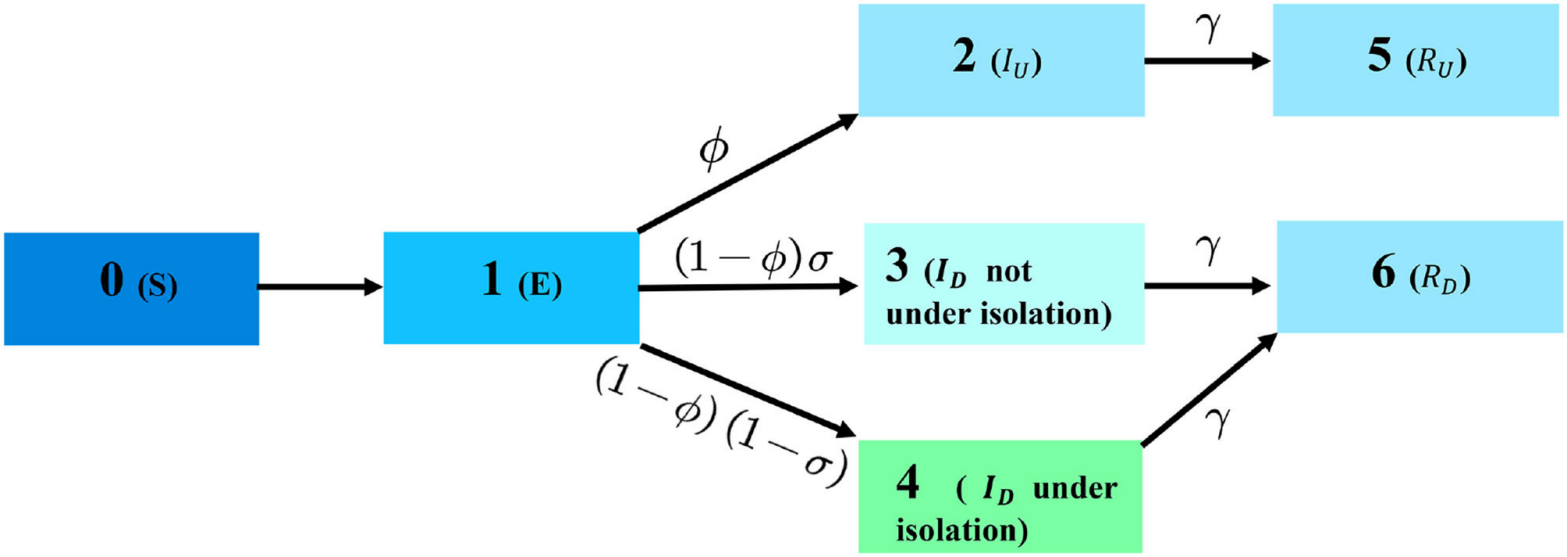
The framework of CA.

The structure of CA is shown in Figure 4.

(a) Expose: If the cell state at *t*−1 was 0 (susceptible), the cell state has a possibility to become 1 (exposed) if any neighbors were 2 or 3 at *t*−1;
(b) Infect into *I*_*U*_: If the cell state at *t*−1 was 1 (exposed), the cell has a chance of ϕ to become 2 if the corresponding number in time matrix, *T*_*i, j*_ is greater than the average confirmed time;
(c) Infect into *I*_*D*_: If the cell state at *t*−1 was 1 (exposed), the cell has a chance of (1−ϕ)σ to become 3 (*I*_*D*_ not under isolation) meantime a probability of (1−ϕ)(1−σ) turn into 4 (*I*_*D*_ under isolation), if the corresponding number in time matrix, *T*_*i, j*_ is greater than average confirmed time;
(d) Recover: If the cell state at *t*−1 was 2, 3, or 4, the cell state becomes 5 (recovered) if the corresponding element in the time matrix, *T*_*i, j*_ is greater than the duration of treatment.
(e) Stay: If the cell state and its corresponding number in *T*_*i, j*_ can not meet any of the transmission rules that were previously defined, the cell state remains the same, and the number in the time matrix, *T*_*i, j*_ will plus a random number from a normal distribution with mean 1 and variance 1.

## 4. Experimental Results and Analysis

### 4.1. Data and Parameters Setting

Similar to previous studies (1, 26, 27), we obtained the COVID-19 epidemic data from the COVID-19 Data Repository managed by the local public health agency. The number of confirmed and recovered cases is updated once a day and includes all provinces of China. In this article, we use the data on COVID-19 in the Heilongjiang and Hebei provinces of China in January 2021 to conduct experiments. These data are shown in Tables 1, 2.

**Table 1:** Daily confirmed and recovered cases of Heilongjiang during the outbreak in January 2021.

**Time (day)**	**Daily confirmed cases**	**Recovered cases**	**Time (day)**	**Daily confirmed cases**	**Recovered cases**
7-Jan	1	0	21-Jan	47	6
8-Jan	0	0	22-Jan	56	8
9-Jan	0	0	23-Jan	29	8
10-Jan	0	0	24-Jan	35	8
11-Jan	1	0	25-Jan	53	9
12-Jan	16	1	26-Jan	29	16
13-Jan	43	1	27-Jan	28	34
14-Jan	43	2	28-Jan	21	56
15-Jan	23	3	29-Jan	27	79
16-Jan	12	3	30-Jan	9	87
17-Jan	7	4	31-Jan	22	99
18-Jan	27	4	1-Feb	8	100
19-Jan	16	5	2-Feb	6	127
20-Jan	68	6	3-Feb	4	170

**Table 2:** Daily confirmed and recovered cases of Hebei during the outbreak in January 2021.

**Time (day)**	**Daily confirmed cases**	**Recovered cases**	**Time (day)**	**Daily confirmed cases**	**Recovered cases**
2-Jan	1	0	16-Jan	72	13
3-Jan	4	0	17-Jan	54	13
4-Jan	14	0	18-Jan	35	17
5-Jan	20	0	19-Jan	19	18
6-Jan	53	0	20-Jan	20	26
7-Jan	33	0	21-Jan	18	39
8-Jan	14	0	22-Jan	15	56
9-Jan	46	0	23-Jan	19	73
10-Jan	82	0	24-Jan	11	115
11-Jan	40	0	25-Jan	5	148
12-Jan	90	0	26-Jan	7	218
13-Jan	81	13	27-Jan	3	275
14-Jan	90	13	28-Jan	1	310
15-Jan	90	13	29-Jan	1	404

According to the previous study (28), the value of the infection rate β in Equations (1) and (2) can be computed as follows:


(4) $$\begin{array}{r} \beta =k\times F \end{array}$$

where *F* represents the number of people that a patient has close contact with. According to the data from the National Health Commission, during the first 15 days of the outbreak, the average number of people a patient has close contact with is 12 per day. Then with the implementation of restrictive measures, *k* drops to 5. The parameter *F* is the median of the time-dependent successful infection rate, *F*(*t*). It can be described as follows:


(5) $$\begin{array}{r} F\left(t\right)=\frac{M_{n}\left(t\right)}{M_{s}\left(t\right)} \end{array}$$

There is an incubation period between getting infected with the virus and being confirmed as infected. Some previous studies have performed a simulation of the incubation period of COVID-19 (28), and the result shows that the median incubation period of COVID-19 is 6 days. So, in this study, the parameters *M*_*n*_(*t*) and *M*_*s*_(*t*) represent the sum of daily confirmed cases and the sum of confirmed cases in 6 days preceding time *t* respectively. We calculate the *F*(*t*) of each day during the outbreak using Equation (5) The parameter *k* is the median of *F*(*t*).

According to the data collected by the National Health Commission of China, the daily successful infection rate, *F*(*t*) is calculated by the data of the COVID-19 outbreak in Hebei Province in January 2021. The results are shown in Table 3.

**Table 3:** The daily successful infection rate of Hebei province during the outbreak in January 2021.

**Time (day)**	***F*** **(*t*)**	**Time (day)**	***F*** **(*t*)**
5-Jan	0.60938	13-Jan	0.20057
6-Jan	0.58974	14-Jan	0.19598
7-Jan	0.44484	15-Jan	0.17769
8-Jan	0.32936	16-Jan	0.14816
9-Jan	0.30050	17-Jan	0.13243
10-Jan	0.29280	18-Jan	0.10498
11-Jan	0.24036	19-Jan	0.08197
12-Jan	0.21479	20-Jan	0.06205

The median of *F*(*t*) is 0.054, so as the value of *F*. Finally, we can calculate the infection rate β:


(6) $$\beta =k\times F=\{\begin{array}{cc} 0.648 & \left(t\leq 15\right)\\ 0.270 & \left(t>15\right) \end{array}$$

According to the law of the PRC on the Prevention and Treatment of Infectious Diseases, the isolation will be immediately implemented once the individual is showing the symptoms of COVID-19, thus the non-isolation rate (σ) is set to 0 in outbreaks that take place in China. The percentage of the asymptomatic individual in all infections (ϕ) is a strongly debated aspect, the value of this parameter shows a great difference in outbreaks of COVID-19 that take place in different areas. This phenomenon may due to the definition of asymptomatic infectious has not reached an international agreement and obstacles to fully understanding the virus (29, 30). In this experiment, we choose 0.59 as the value of ϕ according to the prediction of a China medical team. Based on the recovery data of 364 patients in Mobile cabin hospital (13), the average treatment time *G* is 28.1 days. Thus, we take removal rate γ as a constant during the spread of disease and can be defined as $\gamma =\frac{1}{G}=\frac{1}{28.1}$. Now the value of parameters in SIR has all been set. As mentioned above, the virus has an incubation period of 6 days. In the SEIR model, the transmission rate from exposed to infected η is regarded as a constant and can be defined as $\eta =\frac{1}{6}$.

According to the previous study carried out by (31), the infection rate of close contacts is 0.04. During the outbreak, nucleic acid tests will be carried out every 4 days, therefore, the confirmed time of COVID-19 is set to 4 (9, 16).

In this experiment, we use Matlab to develop these models. Additionally, mean absolute error (MAE) is used to evaluate the performance of these models.

### 4.2. Results and Analysis

#### 4.2.1. Small-Scale Outbreaks in China

**Figure 5:**
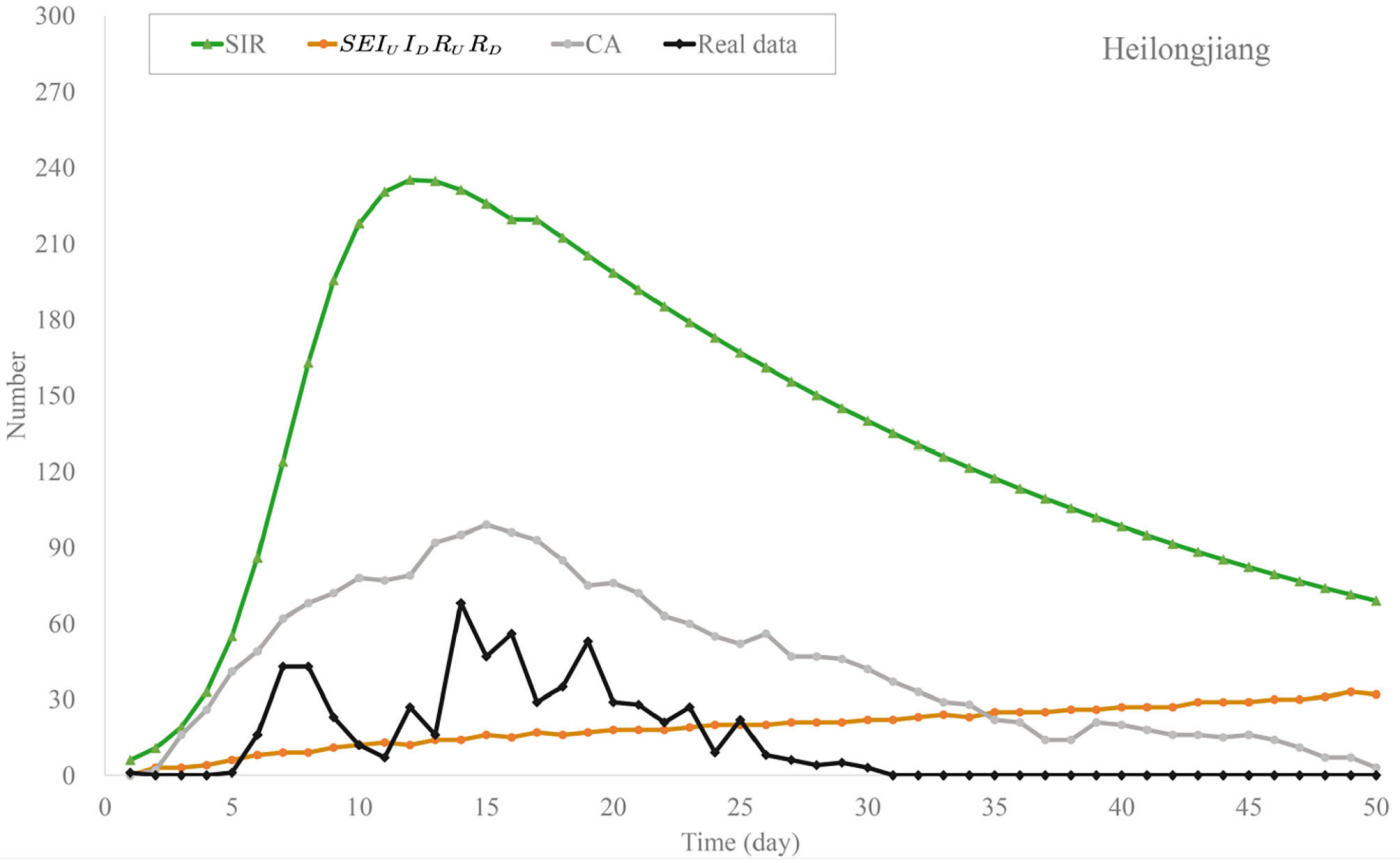
Trend chart of daily confirmed cases of the models in Heilongjiang province.

**Figure 6:**
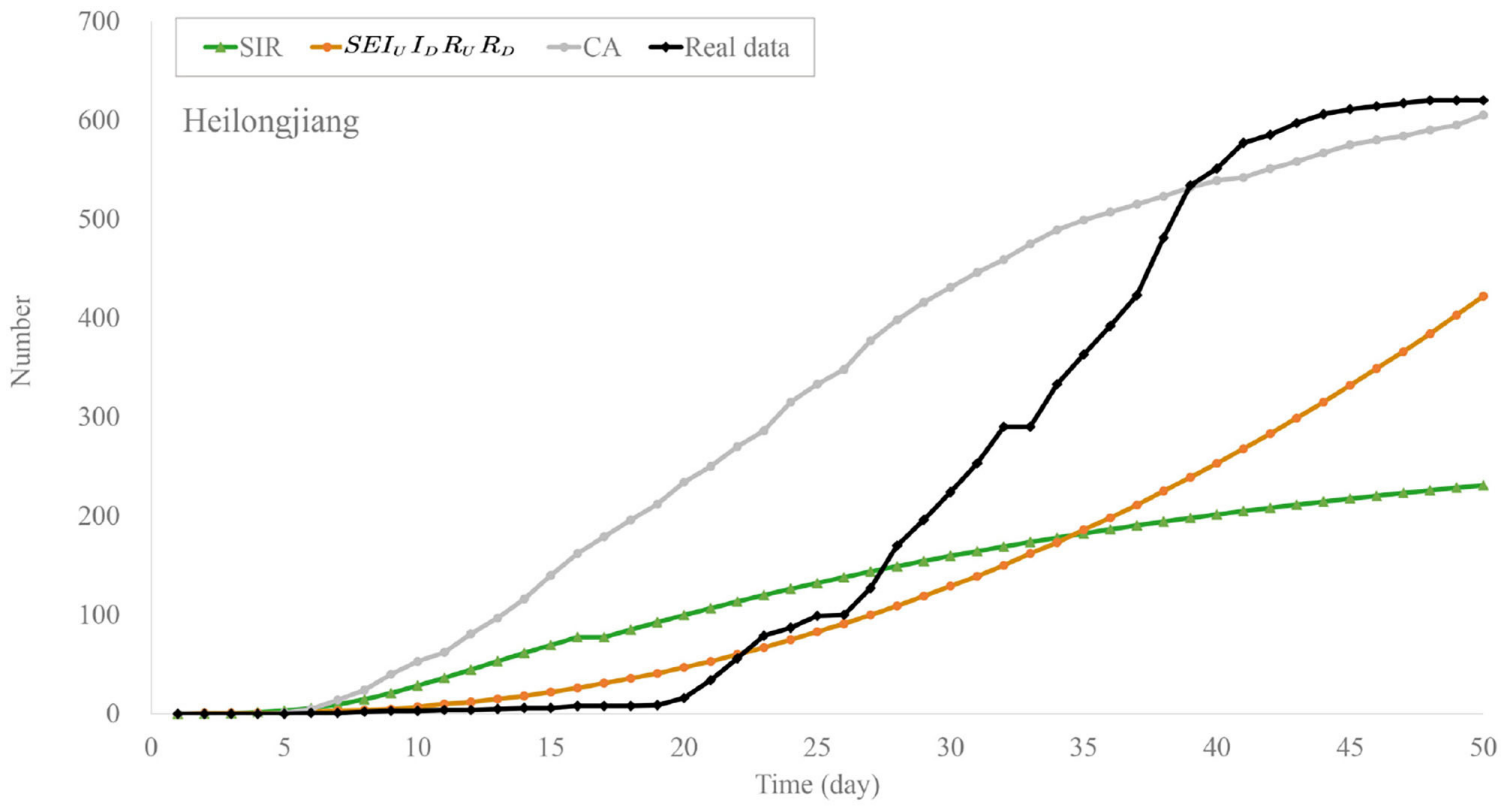
Trend chart of recovered cases of the models in Heilongjiang province.

We predict the outbreaks of COVID-19 in Heilongjiang and Hebei provinces in January 2021. First, Figures 5, 6 show the predicted numbers of the confirmed and recovered cases for Heilongjiang province. These results indicate that CA has a better performance in simulating the outbreak of COVID-19 in China nowadays. Classical compartmental models may no longer be suitable for modeling small-scale outbreaks in China. The MAE values of these methods are shown in Table 4. From these compared results, it can be observed that the proposed CA has the smallest errors. In addition, compared with the traditional CA, the MAE value of the CA model with time matrix is 30.37, which is reduced by 66.35.

**Table 4:** MAE of the models on the outbreak data of Heilongjiang.

**Model**	**MAE**
SIR	122.00
SEIR	95.38
*SEI*_*U*_ *I*_*D*_ *R*_*U*_ *R*_*D*_	37.05
CA without *T*_*i, j*_	99.72
CA	30.37

**Figure 7:**
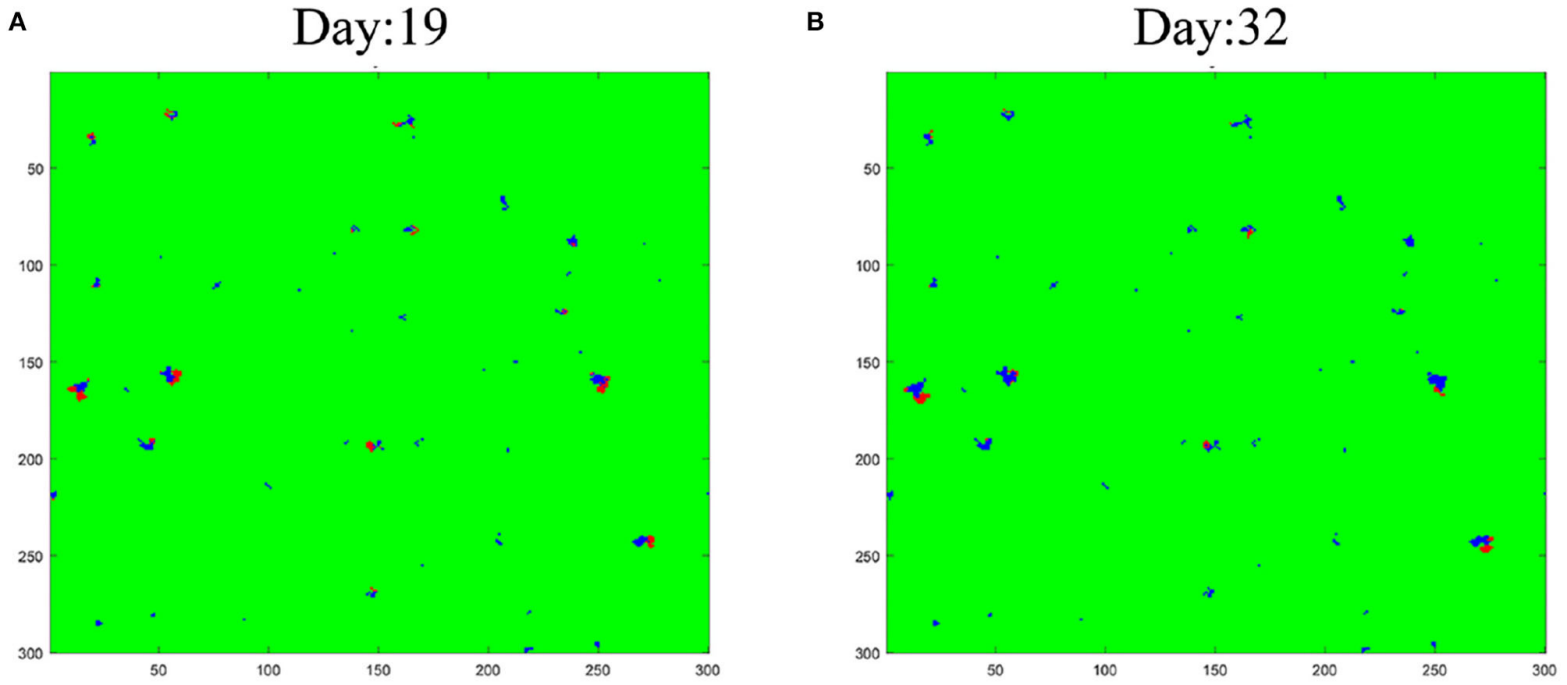
Simulation results of CA: **(A)** is the results of 19th day; **(B)** is the results of 32th day.

We further visualize the simulation results of CA on the 19th day and the 32th day of Heilongjiang province. The red spots represent *I*_*D*_-cell, and the blue is *R*_*D*_-cell. In Figure 7A, there is only a marginal amount of *R*_*D*_-cell and some red spots. In Figure 7B, one can also see that most red spots have turned blue which means that the outbreak is coming to an end. This is consistent with the actual situation of the outbreak in China. The transmission of COVID-19 can get under control within a month. More results about CA can be found in the Supplementary Materials.

**Figure 8:**
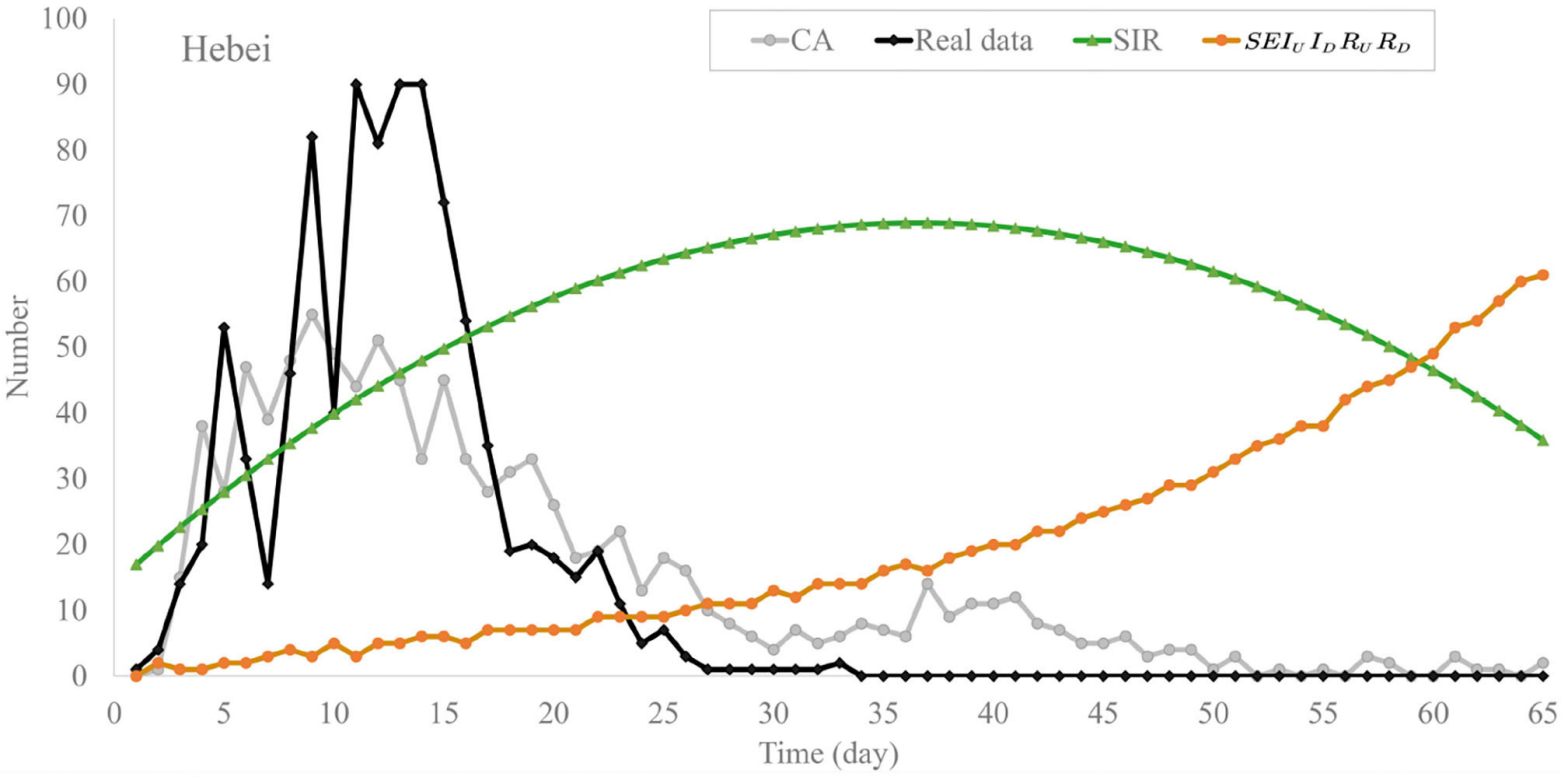
Trend chart of daily confirmed cases of the models in Hebei province.

**Figure 9:**
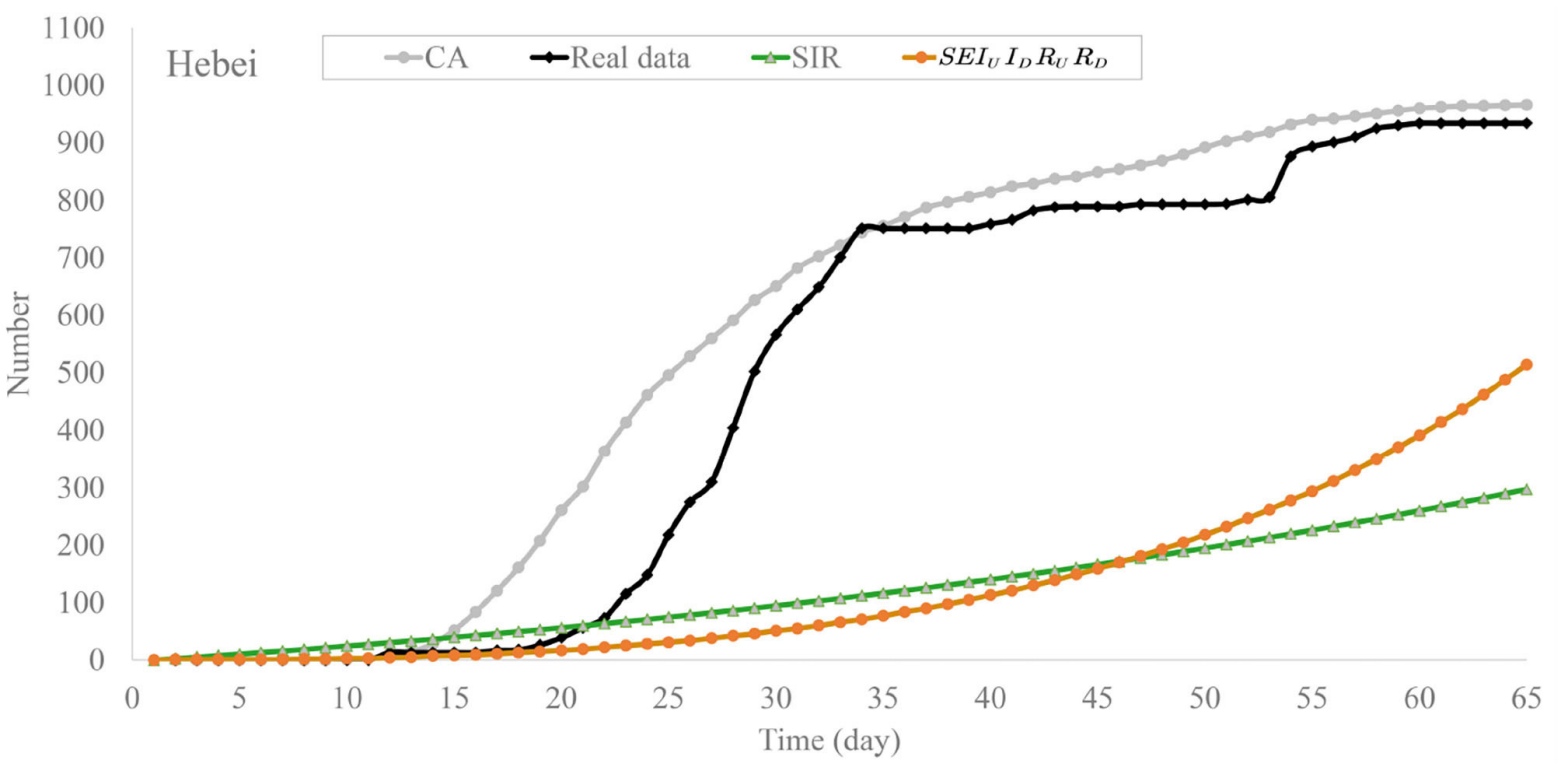
Trend chart of recovered cases of the models in Hebei province.

Once again, we analyzed the data of outbreaks in Hebei province in January 2021 and evacuated these models. The experimental results are shown in Figures 8, 9. In Figure 8, the daily confirmed cases of the SIR model grew rapidly and reached 350 on the 100th day, which makes it deviate from official data. Compared with SIR, the SEIR model has a significant improvement in the fitting, the daily confirmed cases of *SEI*_*U*_*I*_*D*_*R*_*U*_*R*_*D*_ slowly rose to 10 person at 35th day and climbed steadily to 60 at the end of outbreak. Compared with the first two models, the cellular automata can fit in the data with high accuracy. The difference between the maximum time of cellular automata and real data is about 5 days, and the trends of the two curves are roughly the same.

In Figure 9, the recovered cases of SIR keep increasing and reach about 270 on the 65th day. The recovered cases of *SEI*_*U*_*I*_*D*_*R*_*U*_*R*_*D*_ remained at a low value during the former part of outbreak, and grow from around 70 person at 35th day to 500 person at 65th day. We can see that recovered cases of CA are close to 0 from 0 to 18 days then grow dramatically to 800 in the 45th day and slowly climb to around 1,000 in the remaining time. The predicted results of CA are close to the official data.

The MAE values of these methods of the outbreak in Hebei province are shown in Table 5. The MAE of SIR, SEIR, *SEI*_*U*_*I*_*D*_*R*_*U*_*R*_*D*_, CA without *T*_*i, j*_ and CA are 47.98, 44.34, 30.35, 40.93, and 9.43, respectively. It is clear that the MAE of SIR and *SEI*_*U*_*I*_*D*_*R*_*U*_*R*_*D*_ is more than 4 times CA, they performed poorly in both long-term and short-term fitting. These differential models based on compartments may not be suitable for fitting the small-scale outbreaks.

**Table 5:** MAE of the models on the outbreak data of Hebei.

**Model**	**MAE**
SIR	47.98
SEIR	44.34
*SEI*_*U*_ *I*_*D*_ *R*_*U*_ *R*_*D*_	30.35
CA without *T*_*i, j*_	40.93
CA	9.43

#### 4.2.2. The Outbreak in Potter County Texas US

Furthermore, we performed our CA in the simulation of the small-scale outbreak in Potter County, to test its reliability in a different country. Unfortunately, the databases of Potter County health department just maintained confirmed cases and death cases, and the collection of recovered cases has been stopped since April 2021, which means that the, *M*_*s*_(*t*), sum of confirmed cases in 6 days preceding time t are unknown. Because daily recovered cases are not in the record, which makes the existing confirmed cases of each day stay unclear. As a consequence, Equation (4), which defined to calculate the infection rate β, is out of work. In that case, we refer to the previous study and determine the value of infection rate, β, is 0.4428. According to the Centers for Disease Control and Prevention (CDC), the current best estimated average time from exposure to symptom onset is 6 days. Therefore, parameter η = 1/6. The percentage of asymptomatic infections in the US is 30% (ϕ = 0.3). The non-isolation rate of the symptomatic infectious individual (σ) is 0.5. The estimation of the average treatment time is 24.7 (32) $\left(\gamma =\frac{1}{G}=\frac{1}{24.7}\right)$.

**Figure 10:**
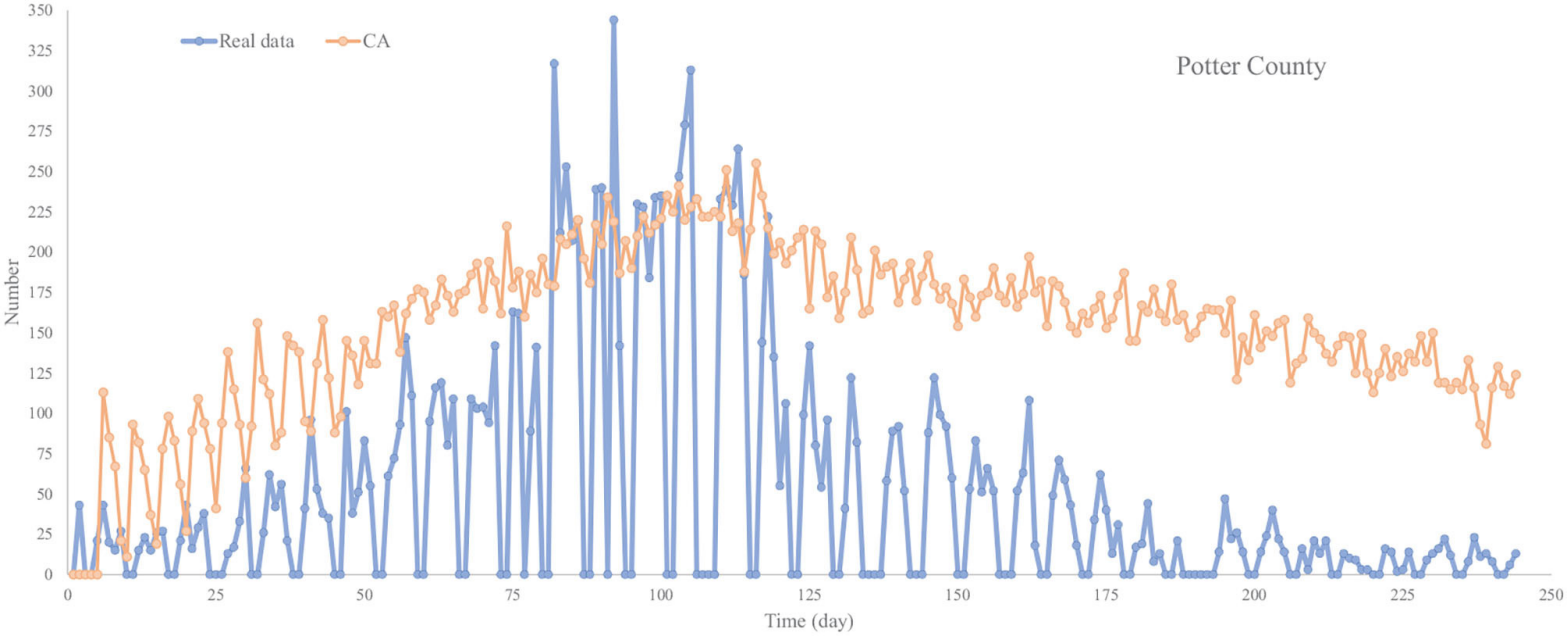
Trend chart of daily confirmed cases of CA in Potter County.

The result of daily new cases can be seen in Figure 10. Table 6 shows the MAE for each model in Potter County. The Figure 10 gives a breakdown of the trend of CA and real data. The real data of daily cases rise dramatically during the former part of the outbreak and reach the beak in around 92 days with 350 new cases. CA performs well in the former part; it also reaches a peak of 250 new cases at around the same time as real data. However, the daily new cases decrease severely to around 100 shortly after the peak, and slowly down to 0 during the later part of the outbreak. The result of CA in the later part of the outbreak is relatively unsatisfactory. It falls to simulate the sharp decrease and there are still around 100 new cases at the end of the outbreak. But on the bright side of our model, it simulates the former part of the outbreak in Potter County with relatively high accuracy, which means that our CA can roughly simulate the trend in small-scale outbreaks outside China.

**Table 6:** MAE for models in Potter County.

**Model**	**MAE**
SIR	201.36
SEIR	92.23
*SEI*_*U*_ *I*_*D*_ *R*_*U*_ *R*_*D*_	96.87
CA	79.25

## 5. Discussion

With appropriate parameters and rules, compared with SIR, SEIR, and *SEI*_*U*_*I*_*D*_*R*_*U*_*R*_*D*_, our CA can simulate the small-scale outbreaks of COVID-19 in nowadays China more effectively. The MAE of CA in the outbreak that took place in Hebei reached a value of 9.43, it provides valuable information about the decision on medical policy. Classic compartmental models have been widely used in modeling the transmission dynamics with numerous infected cases, and have gained great success (7, 33). One major drawback of those compartmental models is the hiking of the number and complexity of parameters (6). The parameters of these models had to be more precise and complex to achieve better performance (26). Although many researchers hold the belief that non-linearities in CA alongside ABM destroyed any attempt to use the predicatively, they are oversimplified from realistic words (34, 35). However, this study has proved that small-scale outbreaks can be modeled through a relatively simple abstract model.

### 5.1. Strengths and Weaknesses

In this contribution, we proposed an improved CA to carry out experiments that introduced a time matrix to have a precise simulation of the outbreak. As the results shown, CA simulated the outbreak accurately which suggests that researchers can consider using it to study the current epidemics in China. However, our rules and parameters of CA are far from perfect. There have been numerous methods to estimate the value of parameters (9, 11, 14). It is true that all parameters are set according to the best simulation rest from the local health department or CDC in the real world situation. But they may not be the perfect values we need according to the structure of our model. During the experiments, we use a time matrix to record the time of virus infection of each cell. Only if the t_(*i, j*)_ is greater than the average treatment time *G* shall the cell state turn into 3, which means that a patient can only recover from COVID-19 after 28 days of treatment. However, this assumption is an oversimplification, as young patients may get recovered before 28 days of treatment, while the aged typically need more time during treatment (9).

### 5.2. Further Study

In further study, the recovered rate γ will no longer be regarded as a constant in CA. At different times of treatment, the recovery rate will be different. Combined with the time matrix, the transition rules of COVID-19 in CA will be updated. In addition, the structure of CA is a 300 square static orthogonal matrix. Each cell is adjacent to 8 others. In further study, the best adjacent number is needed to be determined, since each cell may have interactions with 4, 6, or more neighbors. In the real world, the number of people an individual have contact with is different from each day, as a result, the cell in the CA may intricate with a different number of neighbor each day at further study. Another major improvement in the future is that we will change our CA into an ABM model. Because ABM is developed from CA, they share many similar characters (36, 37). The existing platforms for ABM are fundamentally helpful when setting up transform rules (38–40).

## Data Availability Statement

The original contributions presented in the study are included in the article/Supplementary Material, further inquiries can be directed to the corresponding author/s.

## Author Contributions

Z-YW: methodology and writing—original draft preparation. H-BZ and H-FZ: writing—review and editing. All authors have read and agreed to the published version of the manuscript.

## Funding

This study was supported by the National Key Research and Development Program of China (No. 2019YFC1604700) and the Promotion Program for Young and Middle-Aged Teachers in Science and Technology Research of Huaqiao University (ZQN-YX601).

## Conflict of Interest

The authors declare that the research was conducted in the absence of any commercial or financial relationships that could be construed as a potential conflict of interest.

## Publisher's Note

All claims expressed in this article are solely those of the authors and do not necessarily represent those of their affiliated organizations, or those of the publisher, the editors and the reviewers. Any product that may be evaluated in this article, or claim that may be made by its manufacturer, is not guaranteed or endorsed by the publisher.
