# Supplementary material for: Modeling of the Small-Scale Outbreak of COVID-19
Source: Front Public Health. 2022 Jul 1;10:907814. doi: 10.3389/fpubh.2022.907814 (PMC9283974; doi:10.3389/fpubh.2022.907814)
Supplement: Supplementary file 1 [file Image_1.pdf]

Supplementary Material

Supplementary Figure

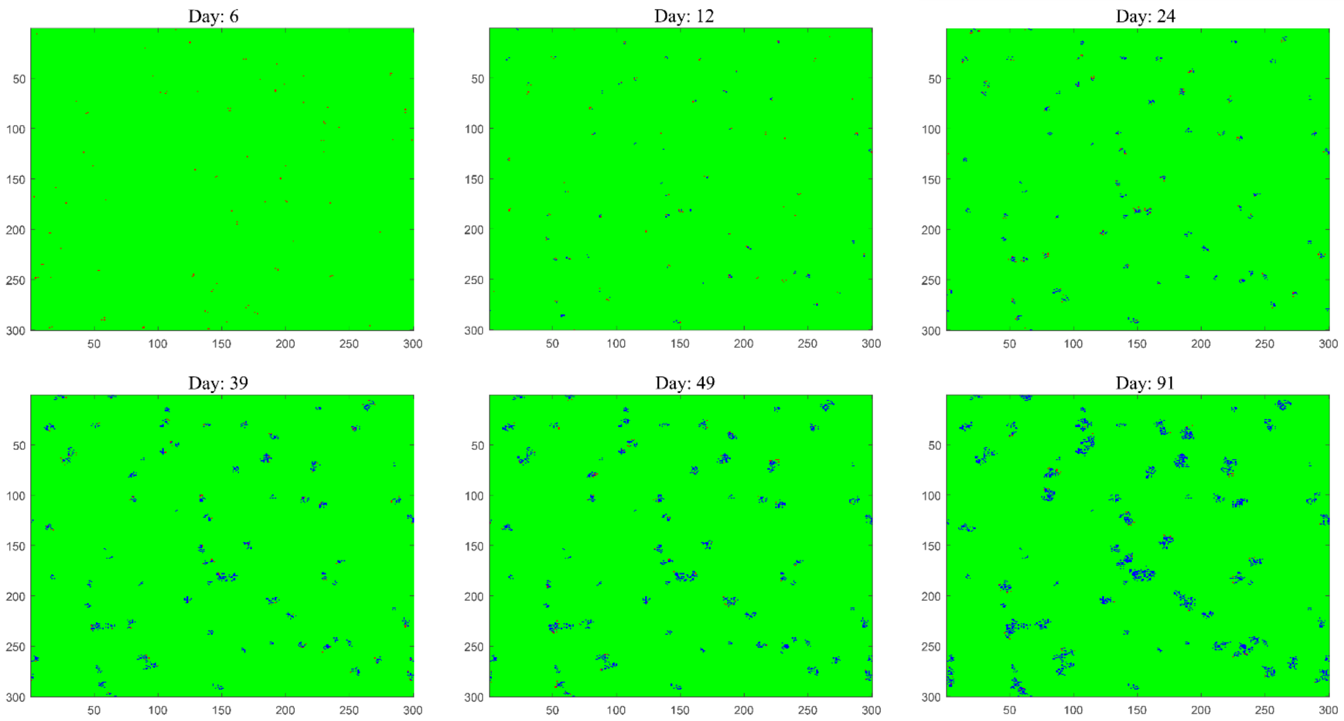

Figure S1. The simulation results of the proposed CA during the outbreak of COVID-19 in Heilongjiang province (January 2021).
